# An efficient quantum partial differential equation solver with chebyshev points

**DOI:** 10.1038/s41598-023-34966-3

**Published:** 2023-05-12

**Authors:** Furkan Oz, Omer San, Kursat Kara

**Affiliations:** School of Mechanical and Aerospace Engineering, Oklahoma State University, Stillwater, OK 74078 USA

**Keywords:** Aerospace engineering, Quantum simulation

## Abstract

Differential equations are the foundation of mathematical models representing the universe’s physics. Hence, it is significant to solve partial and ordinary differential equations, such as Navier–Stokes, heat transfer, convection–diffusion, and wave equations, to model, calculate and simulate the underlying complex physical processes. However, it is challenging to solve coupled nonlinear high dimensional partial differential equations in classical computers because of the vast amount of required resources and time. Quantum computation is one of the most promising methods that enable simulations of more complex problems. One solver developed for quantum computers is the quantum partial differential equation (PDE) solver, which uses the quantum amplitude estimation algorithm (QAEA). This paper proposes an efficient implementation of the QAEA by utilizing Chebyshev points for numerical integration to design robust quantum PDE solvers. A generic ordinary differential equation, a heat equation, and a convection–diffusion equation are solved. The solutions are compared with the available data to demonstrate the effectiveness of the proposed approach. We show that the proposed implementation provides a two-order accuracy increase with a significant reduction in solution time.

## Introduction

Today, computational sciences have become indispensable. The advancement of high-performance computing infrastructure has triggered new questions, and as a result, computational sciences have led to numerous new discoveries. Designed in the early 2000s as a game engine accelerator, Graphical Processing Units (GPUs) have quickly become one of the most important tools in computational science, driven by high performance in artificial intelligence (AI) and machine learning (ML) applications. Today, research labs, funding agencies, startups, and big tech companies are starting to agree that quantum computers could be the next breakthrough in computational science (e.g., see a consensus study report by the National Academies of Science, Engineering, and Medicine^1^).

Many researchers from various fields are considering how to transfer algorithms that are frequently used in classical computers today to quantum computing. To advance the field of quantum computing, it is crucial to answer questions like what kinds of structures or algorithms can be more effective in quantum computing and to which parts of a complicated computational process quantum computing can be applied. For example, on the one hand, we are asking how to adapt widely used algorithms such as FFT, multigrid, or other iterative solvers to quantum computing^2–4^, on the other hand, there is a new interest in how to implement the fastest optimization methods and numerical linear algebra applications^5,6^. Also, in this emerging field, developing a workforce that understands the fundamentals of quantum computing has a very important role. For example, some algorithms that have been idle over time that do not scale up well on classical computers might work very efficiently on quantum computers. Therefore, revisiting many computational techniques or algorithms with quantum computers in mind could lead to new technologies.

Partial differential equations (PDEs) are mathematical models of the universe’s physics. Obtaining analytical solutions to some of the practically important PDEs is unattainable yet. As a field that has led the creation of numerous novel algorithms in the PDE community, fluid dynamics, or more precisely computational fluid dynamics, is one of the most widespread applications of quantum computing^7–9^, along with applications in finance^10^, healthcare^11^, and material science^12^. For example, finding a solution to Navier-Stokes (NS) existence and smoothness problem will receive a million-dollar prize from the Clay Mathematics Institute^13^. Finding new algorithms or utilizing new types of hardware to solve challenging PDEs are active areas of research^7,14–18^. With this in mind, many fluid dynamicists are excited that quantum computing could enable such demanding calculations in the coming years. In a nutshell, when describing the interaction between different scales in fluid dynamics, there is a huge transition from the inertial scale, where large fluid structures are described, to the Kolmogorov scale, where dissipation takes place. If we call the size of a circulation bubble on an airplane wing $\ell$, and take it as a typical unit of inertial scale, we can estimate that it might take many decades of CPU time using one teraflop machines to simulate a simulation box of $(10\ell )^3$ with a moderate Reynolds number of $10^5$ (e.g., see a recent discussion^8^). Even at a higher Reynolds number, the possibility of such a direct numerical simulation with the entire aircraft placed at the center of the simulation domain does not seem feasible unless there is a major breakthrough in computational sciences (e.g., we refer the reader to an estimate^19^ that arrives at CPU time of a fraction of the earth’s age).

The recent advent of quantum computers showed that quantum computing significantly speeds up over the corresponding best classical algorithm. Although there are some studies before the 1990s^20,21^, the first major scientific achievement can be accepted as the Shor quantum computing algorithm for factoring large numbers, which can break popular encryption schemes^22^. The interest in quantum computing is increased even more with the Grover search algorithm that can search unstructured databases with crucial speedup^23^. Subsequently, more studies have been conducted that show a speedup with the quantum computation^24,25^.

The development of quantum computing^26–36^ and the advancement of quantum computers^37–47^ led to the algorithms developed for differential equations. There are several strategies to solve PDEs with quantum computing^19,48–70^. However, this paper focuses on the quantum PDE solver algorithm^19,59,60^ that boosts probabilistic measures. This solver uses an (almost) optimal quantum algorithm^71^ to solve a system of ordinary differential equations (ODEs) obtained from PDEs by discretization. The quantum PDE solver is based on a quantum amplitude estimation algorithm (QAEA)^72^ that estimates the amplitude of a state. The algorithm is used to solve Navier–Stokes equations^59,60^, which play a significant role in the aerospace industry as the governing equations model fluid dynamics and aerodynamics. Subsequently, it is used to solve Burger’s equation^19^, which is extensively used as a benchmark problem for computational fluid dynamics solvers.

As significant progress has been made in quantum computing technologies, in this study, we aim to explore how such a distributor in the information processing paradigm can leverage the current trends in developing quantum algorithms for scientific applications. Specifically, we introduce an efficient quantum PDE solver by defining the Gauss-Lobatto-Chebyshev points^73^ and the cubic-spline interpolation method^74^. Gauss-Lobatto-Chebyshev points are also called Chebyshev extreme points. However, in this paper, it is called Chebyshev points shortly. The new approach is used to solve a generic ODE, the heat equation, and the advection-diffusion equation to show the accuracy of the proposed quantum PDE solver. In our proposed approach, using Chebyshev points reduces the number of evaluations for the oracle function and increases the sampling points used in QAEA. Hence, the accuracy of the solver increases. At the same time, the solution time is decreased significantly. Although far from completeness, we hope our paper sheds light on the prospects of designing emerging quantum PDE solvers as government, public and private sectors face an innovation race on quantum information science and technology.

## Quantum PDE solver

The quantum PDE solver^59,60^ is an approach that utilizes QAEA to solve the underlying system. The first task of the solver is spatial discretization as $x\rightarrow x_{j}\; (1\le j\le m)$ and $u(x,t)\rightarrow u(x_{j},t)\equiv u(j,t)$. The spatial boundary points correspond to the grid points $x_{1}$ and $x_{m}$. It is important to note that time *t* remains a continuous parameter. Once the spatial discretization takes place, the PDEs can be represented by a system of ODEs in the form of:

1 $$\begin{aligned} \frac{du(j,t)}{dt} =f(u(j,t))\, (2\le j\le m-1), \end{aligned}$$

where *f*(*u*(*j*, *t*)) is the driver function. The PDEs are represented by a system of ODEs with Eq. (1), which can be solved with an ODE solver. Herein, a quantum algorithm introduced by Kacewicz^71^ will be introduced to solve a system of ODEs. The algorithm requires a bounded function *A*(*j*, *t*) that approximates the exact solution *u*(*j*, *t*) over the time interval $0\le t\le T$. Both *u*(*j*, *t*) and *A*(*j*, *t*) have to satisfy the initial condition:

2 $$\begin{aligned} u(j,0) = A(j,0) = U_{0}(j). \end{aligned}$$

The driver function *f*(*u*) is assumed to have continuous, bounded derivatives to order *r*, with the $r^{th}$ derivative satisfying the Hölder condition:

3 $$\begin{aligned} \left| \,\,\left. \frac{d^{r}f}{du^{r}}\right| _{u_{1}}-\left. \frac{d^{r}f}{du^{r}}\right| _{u_{2}}\,\, \right| < H\left| u_{1} - u_{2} \right| ^{\rho }, \end{aligned}$$

where, $H>0$ and $0< \rho \le 1$. The driver function’s smoothness is parameterized by $q=r+\rho$, with $q\gg 1$ for smooth functions and $q\sim 1$ for non-smooth functions. Hölder class functions^75,76^ satisfy these conditions and they are elements of the Hölder space $\mathscr {F}^{r,\rho }$.

Kacewicz divides the time interval [0, *T*] into *n* primary subintervals, $T_{i} = [t_{i},t_{i+1}]$. The distance of each subintervals are calculated by $h = T/n$, where $t_{i}=ih\; (0\le i\le n)$. It has to be noted that each primary subinterval $T_{i}$ is related to approximate solution $A_{i}(j,t)$. At this step, $\left\{ y_{i}(j)|\; 0\le i\le n-1 \right\}$ is introduced to provide the initial condition for the primary subinterval $T_{i}$: $A_{i}(j,t_{i}) \equiv y_{i}(j)$. The discussion of the $\left\{ y_{i}(j) \right\}$ will be provided in the following equations. Kacewicz further divides each primary subinterval $T_{i}$ to secondary subintervals (sub-subintervals) $t_{i,m}=t_{i}+m\bar{h}$ $(0\le m\le N_{k})$, where $\bar{h}= h/N_{k} = T/n^{k}$ and $N_{k}= n^{k-1}$. In this paper, the representation of the $m^{th}$ sub-subinterval in $T_{i}$ is given as $T_{i,m}=[t_{i,m},t_{i,m+1}]$, and the approximate solution within $T_{i,m}$ is given as $A_{i,m}(j,t)$. The notation $T_{i,m}$ is used for the time interval and it uses uppercase *T*. The lowercase *t* corresponds to the time value. Since the latter always appears with a lowercase letter *t*, the distinction is clear from the context. In order to find the approximate solution, Taylor’s method^77–79^ is used about $t_{i,m}$ as:

4 $$\begin{aligned} A_{i,m}(j,t) = A_{i,m}(j,t_{i,m}) +\sum \limits _{\nu = 1}^{r}\, \frac{1}{\nu !}\, \frac{d^{\nu -1}f(j,t_{i,m})}{dt^{\nu -1}}\left( t-t_{i,m}\right) ^{\nu } + \mathscr{O}\left( \bar{h}^{r+1}\right) . \end{aligned}$$

For Hölder class functions $f\in \mathscr{F}^{r,\rho }$, the parameter *r* is given. For a quasi-smooth driver function *f*(*u*), the parameter *r* is chosen in such a way that the error $\mathscr {O}(\bar{h}^{r+1})$ is sufficiently small. In this study, the order of accuracy is limited to the second order. There are two reasons for this selection: (i) to minimize the time required for the discretization and (ii) to avoid spatial discretization error-dominated solutions. If the error is dominated by spatial error, the accuracy advantage of the present implementation may be negligible. Nonetheless, the solution time advantage will not be affected by any type of error. The approximate solutions $\{A_{i,m}(j,t)\}$ are required to be continuous at the intermediate times $t_{i,m}$: $A_{i,m}(j,t_{i,m+1}) = A_{i,m+1}(j,t_{i,m+1})$. As noted earlier, $\{ y_{i}(j)\}$ requires to provide the initial condition for the approximate solution $A_{i}(j,t)$ for the $\mathrm {i^{th}}$ primary subinterval $T_{i}$. Thus, at $t=t_{i} \equiv t_{i,0}$, it is required that: $A_{i}(j,t_{i})\equiv A_{i,0}(j,t_{i,0}) = y_{i}(j)$. These two requirements determine $A_{i}(j,t)$ throughout the subinterval $T_{i}$. Specifically, if $t\in T_{i,m}$, then $A_{i}(j,t)=A_{i,m}(j,t)$. Once the $\{ A_{i}(j,t)|\; 0\le i\le n-1 \}$ are known, the global, approximate solution is known: $A(j,t) = A_{i}(j,t)$ for $t\in T_{i}$. At this step, for known parameters n, k, and $\{ y_{i}(j)|\; 0\le i\le n-1 \}$, approximate solution *A*(*j*, *t*) can be determined. The details of how the parameters are calculated will be discussed later. Herein, how the $\{ y_{i}(j) \}$ are chosen is explained. In order to calculate $\{ y_{i}(j) \}$, Eq. (1) is integrated over $T_{i}$:

5 $$\begin{aligned} u(j,t_{i+1}) = u(j,t_{i}) +\sum \limits _{m=0}^{N_k-1} \int \limits _{t_{i,m}}^{t_{i,m+1}} d\tau f(A_{i,m}(j,\tau )) +\sum \limits _{m=0}^{N_k-1} \int \limits _{t_{i,m}}^{t_{i,m+1}} d\tau \left[ f(u(j,\tau ))-f(A_{i,m}(j,\tau ))\right] . \end{aligned}$$

It has to be noted the second term has been added and subtracted. As a result, Eq. (5) is exact. To obtain an equation that relates the $\{ y_{i}(j) \}$, Kacewicz replaces $u(j,t_{i})\approx A_{i}(j,t_{i})\equiv y_{i}(j)$ with $y_{i}(j)$; discards the third term on the RHS as it is $\mathscr {O}(\bar{h}^{r+1})$; and writes $\tau = \bar{h}z$ so that Eq. (5) becomes:

6 $$\begin{aligned} y_{i+1}(j)=y_i(j)+N_k\sum \limits _{m=0}^{N_k-1}\frac{\bar{h}}{N_k} \int \limits _{0}^{1} dz\; f(A_{i,m}(j,z)), \end{aligned}$$

for $0\le i\le n-2$. Eq. (6) determines $y_{i+1}(j)$ from $y_{i}(j)$ and the Taylor polynomials $\{ A_{i,m}(j,t) \}$. The $\{ y_{i}(j) \}$ are determined iteratively. The first step sets $y_{0}(j)$ equal to the initial condition: $y_{0}(j) = U_{0}(j)$. The $\{ y_{0}(j) \}$ then determine $A_{0}(j,t)$ throughout the primary subinterval $T_{0}=[0,t_{1}]$ as described above. Eq. (6) then determines $y_{1}(j)$ from $y_{0}(j)$, once the integral on the RHS is evaluated. To that end, Kacewicz introduces $N_{k}$ knot times $\{ z_{m,p} \}$ in each secondary subinterval $T_{i,m}$ and approximates the integral by its average value over the knot times:

7 $$\begin{aligned} \sum \limits _{m=0}^{N_k-1}\frac{\bar{h}}{N_k} \int \limits _{0}^{1} dz f(A_{i,m}(j,z))=\frac{\bar{h}}{N_k^2}\sum \limits _{m,p=0}^{N_k-1}f(A_{i,m}(j,z_{m,p})). \end{aligned}$$

The average value of *f* on the right-hand side of Eq. (7) is calculated by the Quantum Amplitude Estimation Algorithm^72^ (QAEA). However, in this approach, the number of knot points scales up quickly, leading to a slower solution time. To avoid this issue, the Chebyshev points will be introduced to approximate the integral.

### Chebyshev points

Chebyshev points are the roots of the Chebyshev polynomials of the first kind. They are widely used in numerical methods to avoid non-physical oscillations called Runge’s phenomenon^80,81^. In this study, Chebyshev points are utilized to decrease the number of knot points in the algorithm. It has to be noted that other point distributions are available in the literature. However, the Chebyshev points are robust for such an application. In this approach, instead of $N_{k}$ knot times, $K_{nf}$ number of knot points $\{ w_{m,p} \}$ are introduced with Chebyshev points in each sub-subinterval $T_{i,m}$ as:

8 $$\begin{aligned} w_{m,p} = \frac{\cos \left( \frac{\pi p}{K_{nf}-1}\right) +1}{2}\bar{h}+t_{i,m}\;\;\;(0\le p < K_{nf}), \end{aligned}$$

where the number of knot points is much less than the previously introduced number of sub-subintervals ($K_{nf}\ll N_k$). To that end, a new function $q_{m}(g_s)$, which satisfies $q_{m}(g_s)=A_{i,m}(j,w_{m,p})$ at $0\le p < K_{nf}$ is defined in the interval of $[t_{i,m},t_{i,m+1}]$ as:

9 $$\begin{aligned} q_{m}(g_s)=\frac{q''(w_{m,p})}{w_{m,p}-w_{m,p+1}}\frac{(g_s-w_{m,p+1})^3}{6}+\frac{q''(w_{m,p+1})}{w_{m,p+1}-w_{m,p}}\frac{(g_s-w_{m,p})^3}{6}+C\;g_s+D\;\;\;(0\le s < K_{ns}), \end{aligned}$$

where $g_s\in [t_{i,m},t_{i,m+1}]$, *C* and *D* are unknown coefficients. *C* and *D* can be found by matching the functional values at end points^73^. At this step, $K_{ns}$ number of knot points are introduced in the same interval. It has to be noted that $K_{nf}\ll N_k \ll K_{ns}$. Once the new knot points are substituted into Eq. (7), the final equation will be:

10 $$\begin{aligned} \sum \limits _{m=0}^{N_k-1}\frac{\bar{h}}{N_k} \int \limits _{0}^{1} dz f(A_{i,m}(j,z))=\frac{\bar{h}}{K_{nf}}\frac{1}{K_{ns}}\sum \limits _{m=0}^{K_{nf}-1}\sum \limits _{s=0}^{K_{ns}-1}f(q_{m}(g_s)). \end{aligned}$$

In the present implementation, a small amount of discretization is required as $K_{nf}\ll N_k$. Additionally, in the calculation of *f* appearing on the RHS of Eq. (10), a high number of points will be used in the approximation of the integral as $N_k \ll K_{ns}$. This will ensure that the number of calculations in the Quantum Amplitude Estimation Algorithm is higher with the present implementation. As a result, it may lead to a more accurate approximation. It has to be noted that the Chebyshev points are utilized in time integration. Hence, the proposed time integration approach is not affected by the spatial dimension of the problem. While we demonstrated our method on a one-dimensional problem, our proof of concept results indicates that our approach can be easily extended to higher dimensions and more complex domains. In other words, the algorithm can be used in two- or three-dimensional problems. The driver function appearing in Eq. (1) will be the only parameter changing with the dimensionality. Finally, before the QAEA can be used to evaluate Eq. (10), *f* must be shifted and rescaled in such a way that it will be in the range of [0, 1]. Novak^82^ and Heinrich^83^ showed how the QAEA could be used to evaluate a function average, and Gaitan^59,60^ explains how the shift and rescaling are implemented. In this way, the $\{ y_{1}(j) \}$ are determined. They, in turn, determine the $\{ A_{1,m}(j,t)\}$ throughout $T_{1} = [t_{1},t_{2}]$ as described above. This allows the RHS of Eq. (6) to be evaluated using the QAEA to approximate the integral giving the $\{ y_{2}(j) \}$. Iterating this procedure over the remaining primary subintervals $T_{i}$ gives the approximate solution *A*(*j*, *t*), where $A(j,t) = A_{i}(j,t)$ for $t\in T_{i}$ and $0\le i \le n-1$. Kacewicz^71^ shows that for Hölder class functions the solution error $\varepsilon$ satisfies (for $n\ge 5$):

11 $$\begin{aligned} \varepsilon \equiv sup_{\{ j,t\}}\left| u(j,t)-A(j,t) \right| = \mathscr {O}\left( \frac{1}{n^{\alpha _{k}}} \right) , \end{aligned}$$

with probability $1-\delta$. Here $\alpha _{k} = k(q+1)-1$ and $q = r+\rho$ is the driver function smoothness parameter. Vanquez and Woerner^84^ show how error scales with Oracle calls, and Gaitan^59,60^ and Oz^19^ show the complexity analysis of the quantum PDE algorithm by including QAEA.

### Quantum amplitude amplification algorithm

**Figure 1:**
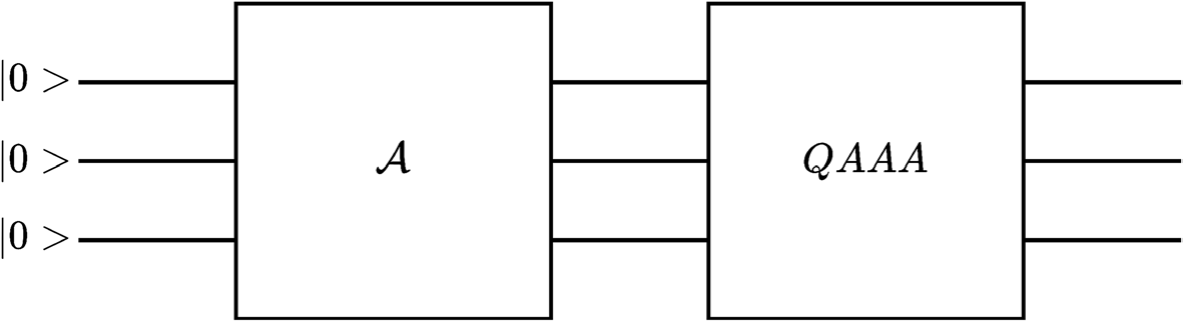
The quantum circuit representation of Quantum Amplitude Amplification Algorithm for three qubits.

Quantum Amplitude Amplification Algorithm (QAAA) is a quantum algorithm that allows finding a desired state with amplified probability, and it is an important part of the quantum PDE solver. The algorithm details are available in Brassard et al.^72^. Moreover, Gaitan^59^ explained the usage of QAAA in the quantum PDE solver. However, we will provide a short explanation of the algorithm for a clear understanding of this study. The quantum circuit for the algorithm is illustrated in Fig. 1.

QAAA starts by introducing unitary operator $\mathscr {A}$ that acts on Hilbert space $\mathscr {H}$ without any measurements. The state obtained by applying $\mathscr {A}$ to zero state is defined as:

12 $$\begin{aligned} |\phi>=\mathscr {A}|0>=|\phi _0>+|\phi _1>. \end{aligned}$$

If we represent Eq. (12) in an alternative form by defining $|n_0>=|\phi _0>/\sqrt{<\phi _0|\phi _0>}$, $|n_1>=|\phi _1>/\sqrt{<\phi _1|\phi _1>}$, and $a=<\phi _1|\phi _1>$. The new equation will be:

13 $$\begin{aligned} |\phi>=\sqrt{1-a}|n_0>+\sqrt{a}|n_1>. \end{aligned}$$

Herein, $|n_1>$ is, following Brassard et al.^72^ terminology, the good (desired) state, and $\sqrt{a}$ is the amplitude of this state. The objective of the QAAA is to amplify this amplitude to obtain a good state. For the amplification, a unitary operator is required, which can be defined as:

14 $$\begin{aligned} Q=-\mathscr {A}S_0\mathscr {A}^{-1}S_{\chi }, \end{aligned}$$

where $S_{\chi }$ is an operator that conditionally changes the sign of the amplitudes of the good states, and $S_0$ is an operator that changes the sign of the amplitude if and only if the state is a zero state. The simple action of *Q* on the subspace $\mathscr {H}_{\phi }$ spanned by the vectors $|\phi _1>$ and $|\phi _2>$ can be shown as:

15 $$\begin{aligned} Q|\phi _1>&=(1-2a)|\phi _1>-2a|\phi _0>\end{aligned}$$

16 $$\begin{aligned} Q|\phi _0>&=2(1-a)|\phi _1>+(1-2a)|\phi _0>, \end{aligned}$$

where $a=<\phi _1|\phi _1>$. If we assume $0<a<1$, the eigenvalues of the operator *Q* will be:

17 $$\begin{aligned} \lambda _\pm =e^{\pm i2\theta _a}, \end{aligned}$$

where $\theta _a$ is defined as:

18 $$\begin{aligned} \sin ^2(\theta _a)=a=<\phi _1|\phi _1>. \end{aligned}$$

Lastly, if we apply operator *Q* *j* times, the final state will be:

19 $$\begin{aligned} Q^j|\phi>=\frac{1}{\sqrt{a}}\sin ((2j+1)\theta _a)|\phi _1>+\frac{1}{\sqrt{1-a}}\cos ((2j+1)\theta _a)|\phi _0>. \end{aligned}$$

Herein, if we choose a *j* in such a way that $\sin ^2((2j+1)\theta _a)$ is close to 1, the amplitude will be greatly amplified for the good state.

### Quantum amplitude estimation algorithm

**Figure 2:**
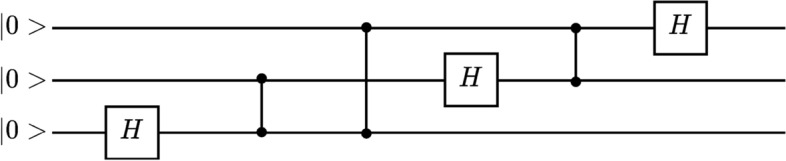
The quantum circuit representation of Quantum Fourier Transform with three qubits without qubit swapping at the end.

Quantum Amplitude Estimation Algorithm (QAEA) is a quantum algorithm that returns an estimate of the quantum amplitude $\sin (\theta _a)$. The algorithm is defined in Hilbert space, $\mathscr {H}'$, with two *n*-qubits registers. It is based on Quantum Phase Estimation^85^ (QPE); however, QAEA differs from QPE with minor changes. The first task in QAEA is to initialize qubits to the state of:

20 $$\begin{aligned} |\Gamma> = |0> \bigotimes |\phi >, \end{aligned}$$

where $|\phi >=\mathscr {A}|0>$. After that, Quantum Fourier Transform (QFT) can be applied to the first register. The representative circuit of QFT is illustrated in Fig. 2.

**Figure 3:**
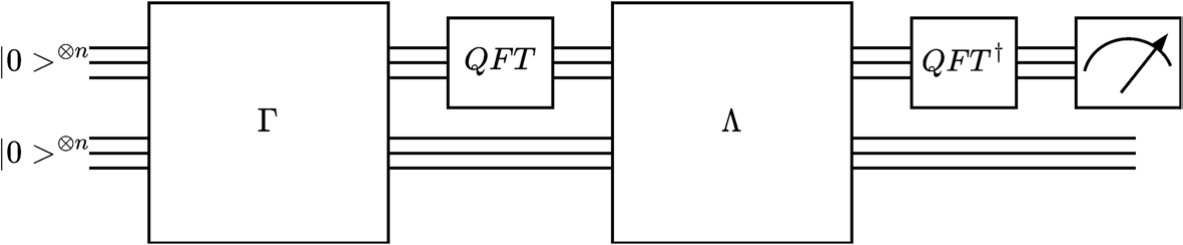
The quantum circuit representation for Quantum Amplitude Estimation Algorithm.

In the next step, a new operator whose action on the computational basis is defined as:

21 $$\begin{aligned} \Lambda (Q)|l> \bigotimes |m> = |l> \bigotimes Q^l|m>, \end{aligned}$$

where $|l>\bigotimes |m>$ are states defined in Hilbert space, $\mathscr {H}'$. Finally, the algorithm requires applying inverse QFT (QFT$^\dagger$) to the first register and measuring it. When the measurement, *M*, is substituted into equation $\tilde{\theta _a}=\pi M/N$, the approximate value of *a* can be calculated. Herein, *N* is defined as $N=2^n$. The circuit representation of the algorithm is given in Fig. 3. This algorithm summarizes the QAEA. For further details, Novak^82^ implemented QAEA to calculate the function mean value, and Gaitan^59,60^ developed a quantum PDE solver by calculating the function mean value with QAEA.

## Governing equations

The quantum PDE solver starts by discretization to convert PDEs into a system of ODEs. This paper will use three test cases: a generic ODE, heat equation, and convection-diffusion equation. The cases include several vital terms of complex PDEs. The generic ODE case does not require any spatial discretization. Hence the numerical error will reveal the advantage of the current implementation. The heat and convection–diffusion equations include important terms of Navier-Stokes equations, which are the fundamental equations of the aerospace industry.

### Ordinary differential equation

In principle, the quantum PDE solver^59,60^ converts a PDE into a system of ODEs to integrate over time (Eq. (1)). In this procedure, the discretization of PDEs introduces spatial numerical error to the system. To avoid and differentiate the error stemming from the spatial discretization, a generic ODE will be integrated with the proposed quantum PDE solver. The ODE used as a test case is:

22 $$\begin{aligned} \frac{d u}{d t}=\frac{e^{t/10}}{10}+\sin (t)+4\sin (2t)+3\sin (3t)+4\sin (4t)-5\sin (5t)-6\sin (6t)-7\sin (7t), \end{aligned}$$

where the analytical solution can be obtained by integrating Eq. (22) as:

23 $$\begin{aligned} u(t)=e^{t/10}-\cos (t)-2\cos (2t)-\cos (3t)-\cos (4t)+\cos (5t)+\cos (6t)+\cos (7t). \end{aligned}$$

The ODE includes Sine function terms with several frequencies and an exponential term. While different frequencies contribute to the complexity of the problem, the exponential term increases the amplitude. Additionally, the ODE does not require to be spatially discretized because it is already in the form given in Eq. (1). The simulation details and initial conditions used in the numerical experimentation will be detailed later when we present our results.

### Heat equation

The heat equation in one physical dimension can be written as:

24 $$\begin{aligned} \frac{\partial u}{\partial t} =\alpha ^2 \frac{\partial ^2 u}{\partial x^2}, \end{aligned}$$

where *t* is the time, $\alpha ^2$ is the thermal diffusivity. The quantity of interest for which the equation is solved is referred to as *u*. The boundary and initial conditions of the equation are:

25 $$\begin{aligned} u(x,0)=f(x)=\sin (\pi x)\end{aligned}$$

26 $$\begin{aligned} u(0,t)=u(L,t)=0, \end{aligned}$$

where *L* is the length of the spatial interval. The analytical solution of the equation can be obtained by the separation of variables method^86^. For the given boundary and initial conditions, the analytical solution can be obtained as follows:

27 $$\begin{aligned} u(x,t)&=\sum \limits _{k=1}^\infty \beta _k \sin \left( \frac{k\pi }{L}x\right) e^{-\alpha ^2\pi ^2k^2t/L^2}&k&=1,2,3,...,\infty \end{aligned}$$

28 $$\begin{aligned} \beta _k&=\frac{2}{L}\int \limits _0^Lf(x)\sin \left( \frac{k\pi x}{L}\right) dx&k&=1,2,3,...,\infty . \end{aligned}$$

where *f*(*x*) is the initial condition given in Eq. (25), the subscript *k* corresponds to indices in Fourier sine series and *L* is the length of the spatial interval. *L* is taken as 1 in this study. Since the initial condition is a sine function and the sine is an orthogonal function, the final analytical solution will be:

29 $$\begin{aligned} u(x,t)=\sin (\pi x)e^{-\alpha ^2\pi ^2t}. \end{aligned}$$

The heat equation is a PDE that needs to be discretized to convert the system in the form of Eq. (1). The system of ODEs for heat equation is:

30 $$\begin{aligned} \frac{du(j,t)}{dt}={}&\frac{\alpha ^2}{12\Delta x^2}[+11u(j-1,t)-20u(j,t)+6u(j+1,t)+4u(j+2,t)-u(j+3,t)] \;\;\;\; (j=2) \end{aligned}$$

31 $$\begin{aligned} \frac{du(j,t)}{dt}={}&\frac{\alpha ^2}{12\Delta x^2}[-u(j-2,t)+16u(j-1,t)-30u(j,t)+16u(j+1,t)-u(j+2,t)] \;\;\;\; (2< j< m-1) \end{aligned}$$

32 $$\begin{aligned} \frac{du(j,t)}{dt}={}&\frac{\alpha ^2}{12\Delta x^2}[-u(j-3,t)+4u(j-2,t)+6u(j-1,t)-20u(j,t)+11u(j+1,t)]\;\;\;\; (j=m-1), \end{aligned}$$

where the lattice spacing $\Delta x = x_{j}-x_{j-1}$ is assumed to be constant. The fourth-order finite difference scheme is used as a method for spatial derivative. The reason a high-order scheme is used is to decrease the spatial error to reveal the accuracy improvements with the new implementation. Otherwise, spatial error dominates the total error, and accuracy comparison becomes misleading.

### Convection–diffusion equation

The convection–diffusion equation in one dimension can be written as:

33 $$\begin{aligned} \frac{\partial u}{\partial t}+c\frac{\partial u}{\partial x}=\alpha ^2\frac{\partial ^2 u}{\partial x^2}, \end{aligned}$$

where *t* is the time, $\alpha ^2$ is the thermal diffusivity, *c* is the velocity of the wave, and *u* is the parameter that the equation is solved for. The initial conditions of the equation can be given by:

34 $$\begin{aligned} u(x,0)=f(x)=\sin (2\pi x). \end{aligned}$$

For this test case, a periodic boundary condition is used. The analytical solution of the equation can be solved with the separation of variables method as the heat equation. The final analytical solution for given initial and boundary conditions reduces:

35 $$\begin{aligned} u(x,t)=\sin (2\pi (x-ct))e^{-\alpha ^2 4\pi ^2 t}. \end{aligned}$$

The convection–diffusion equation must also be written as Eq. (1). The system of ODEs for the convection–diffusion equation is:

36 $$\begin{aligned} \frac{du(j,t)}{dt}={}&\frac{-c}{12\Delta x}[u(m-2,t)-8u(m-1,t)+8u(j+1,t)-u(j+2,t)]\nonumber \\&\frac{\alpha ^2}{12\Delta x^2}[-u(m-2,t)+16u(m-1,t)-30u(j,t)+16u(j+1,t)-u(j+2,t)] \;\;\;\; (j=1) \end{aligned}$$

37 $$\begin{aligned} \frac{du(j,t)}{dt}={}&\frac{-c}{12\Delta x}[u(m-1,t)-8u(j-1,t)+8u(j+1,t)-u(j+2,t)]\nonumber \\&\frac{\alpha ^2}{12\Delta x^2}[-u(m-1,t)+16u(j-1,t)-30u(j,t)+16u(j+1,t)-u(j+2,t)] \;\;\;\; (j=2) \end{aligned}$$

38 $$\begin{aligned} \frac{du(j,t)}{dt}={}&\frac{-c}{12\Delta x}[u(j-2,t)-8u(j-1,t)+8u(j+1,t)-u(j+2,t)]\nonumber \\&\frac{\alpha ^2}{12\Delta x^2}[-u(j-2,t)+16u(j-1,t)-30u(j,t)+16u(j+1,t)-u(j+2,t)] \;\;\;\; (2< j< m-1) \end{aligned}$$

39 $$\begin{aligned} \frac{du(j,t)}{dt}={}&\frac{-c}{12\Delta x}[u(j-2,t)-8u(j-1,t)+8u(j+1,t)-u(2,t)]\nonumber \\&\frac{\alpha ^2}{12\Delta x^2}[-u(j-2,t)+16u(j-1,t)-30u(j,t)+16u(j+1,t)-u(2,t)] \;\;\;\; (j=m-1) \end{aligned}$$

40 $$\begin{aligned} \frac{du(j,t)}{dt}={}&\frac{-c}{12\Delta x}[u(j-2,t)-8u(j-1,t)+8u(2,t)-u(3,t)]\nonumber \\&\frac{\alpha ^2}{12\Delta x^2}[-u(j-2,t)+16u(j-1,t)-30u(j,t)+16u(2,t)-u(3,t)] \;\;\;\; (j=m). \end{aligned}$$

The details of the initial and boundary conditions will be provided in the following section.

## Results

In this section, the new implementation will be applied to three different test cases, a generic ordinary differential equation, the heat equation, and the convection–diffusion equation. The results of the present approach will be compared with the analytical solution and available literature^19,59,60^. It is important to state that the quantum PDE solver is developed by Gaitan^59,60^. Oz et al.^19^ implemented the quantum PDE solver to Burgers’ equation (BE). In the present paper, the algorithm utilized in Reference^19^ will be used for the new test cases and compared with the proposed implementation. Moreover, solution time differences will be investigated. Before presenting the results, the common input parameters required in the quantum PDE solver will be introduced. Firstly, the order of the bounded derivatives, *r*, that appears in Eq. (3) is taken as 2. It is a free parameter that affects the order of accuracy of the solver. The impact of the parameter on the solution vector will be discussed later in the paper.

Kacewicz’s quantum algorithm requires specifying the error bound and the probability. The error bound is defined as $\varepsilon _{1} = 0.005$, and the probability $\delta = 0.005$ is the probability that the quantum algorithm returns a solution that violates the bounds of the quantum algorithm. Kacewicz requires $\varepsilon _{1} = 1/n^{k}$. Solving this expression for *k* gives:

41 $$\begin{aligned} k = 1 + \lceil \ln \left( 1/\varepsilon _{1}\right) /\ln (n) \rceil . \end{aligned}$$

The error bound is already specified. However, *n* and *k* are still free parameters. To that end, Courant-Friedrichs-Lewy (CFL) stability condition^87^ is introduced in the old implementation, and a value is specified in such a way that the stability conditions meet. It ensures that there will not be instabilities developing because of the time discretization. In the present method, the number of intervals *n* is the free parameter, and *k* will be calculated with Eq. (41). Later, the free parameter *n* will be changed to investigate the accuracy and solution time differences between the two implementations. It has to be noted that *n* has to be within the stability limit to converge a solution. Otherwise, the simulation will diverge.

### Ordinary differential equations

An ordinary differential equation is defined in Eq. (22) with the analytical solution given in Eq. (23). The given ODE has sine functions with various frequencies and an exponential function. The discretization of the equation lacks lattice spacing. Thus, the error does not include spatial discretization error.

**Figure 4:**
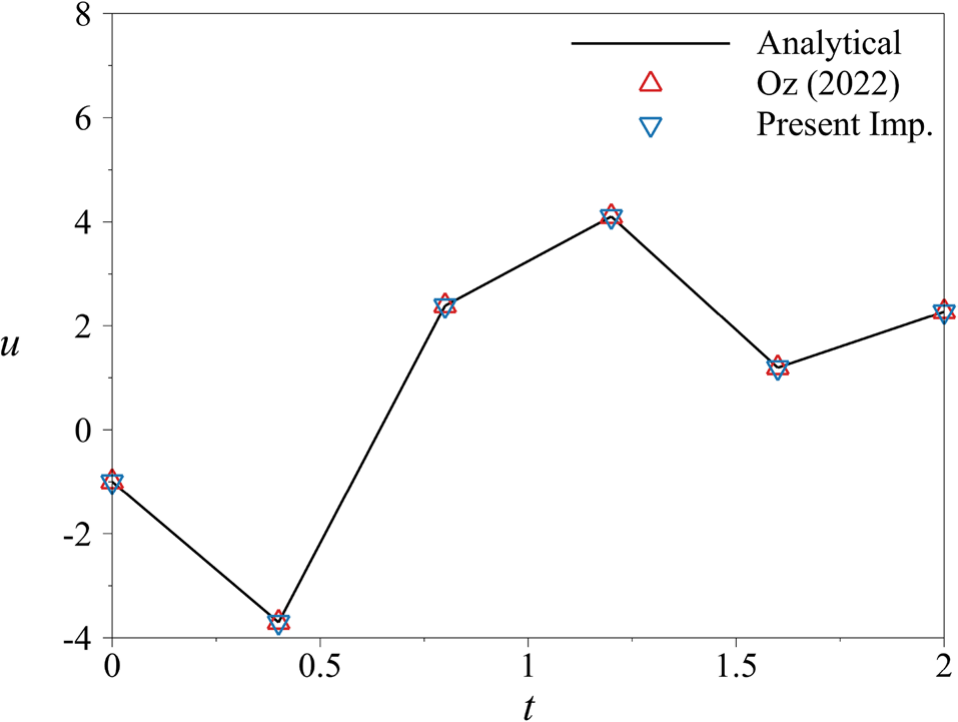
The comparison of results obtained with quantum PDE solver using two implementations and the analytical solution in the interval of $T=[0\;2]$ with $n=5$. The results show no distinct difference.

In the simulation, the initial condition is taken as $u(0)=-1$, which is calculated from the exact solution at the initial time, and the simulation is run in the interval of $T = [0\;2]$. As previously stated, this problem is an initial value problem. Therefore, there is no boundary condition for *u*(2). First, the simulation is carried out with the implementation used by Oz et al.^19^. The conditions implemented for the number of subintervals *n* led to 5. The number of sub-subintervals and knot points $N_k$ led to 625. In the present implementation, the number of subintervals *n* is a free parameter. However, it is specified as 5 for a better comparison. For this case, $K_{nf}$ is taken as 10 and $K_{ns}$ is taken as 50, 000. As previously stated, $K_{nf}\ll N_k \ll K_{ns}$. Herein, the selection of $K_{nf}$ and $K_{ns}$ is arbitrary. However, results with various selections will be provided later. Figure 4 shows the analytical solution with the comparison of two implementations of quantum PDE solver along with the analytical solution given in Eq. (23). As expected, the results have no distinct difference.

To examine the performance of the new implementation, solution time, L$_1$, L$_2$, and L$_\infty$ errors are calculated. The errors are defined as:

42 $$\begin{aligned} |x|_p=\left( \sum \limits _i |x_i|^p\right) ^{1/p},\;\;\;|x|_{\infty }=max(x_i),\;\;\;(p=1,2),\;(0\le i \le n), \end{aligned}$$

where *n* is the number of subintervals, $x_i=u_{an,i}-u_i$, and $u_{an,i}$ and $u_i$ are the exact solution obtained from the analytical expression and the results obtained from the carried out simulations, respectively. The solution time is normalized with the solution time obtained with the implementation used in the quantum BE solver. It has to be noted that solution times given in this study are to provide an idea about the implementations rather than a benchmark test. Table 1 compares the normalized solution times and errors defined in Eq. (42). The L$_1$ error difference indicates that the summation of the errors at each subinterval improved one order with the present implementation. Moreover, L$_2$ norm, which is extensively used in the literature to show the accuracy of the solutions, and L$_\infty$ norm, which shows the maximum error in the solution vector, is also improved one-order with the present implementation. The present implementation also decreased the solution time by approximately 100 times, which is a drastic decrease with the improved accuracy.

**Table 1:** The normalized solution times and errors obtained by the quantum PDE algorithm for two different implementations.

	Solution	L$_1$	L$_2$	L$_\infty$
	Time	Error	Error	Error
Oz^19^	1	$3.8690\times 10^{-5}$	$2.0905\times 10^{-5}$	$1.7964\times 10^{-5}$
Present Imp.	0.012	$5.6736\times 10^{-6}$	$3.0930\times 10^{-6}$	$2.5859\times 10^{-6}$

We only showed a test case with a constant number of subintervals and sub-subintervals. In the new test case, the number of subintervals and sub-subintervals will be chosen as a free parameter for the implementation used in the quantum BE solver as well.

**Figure 5:**
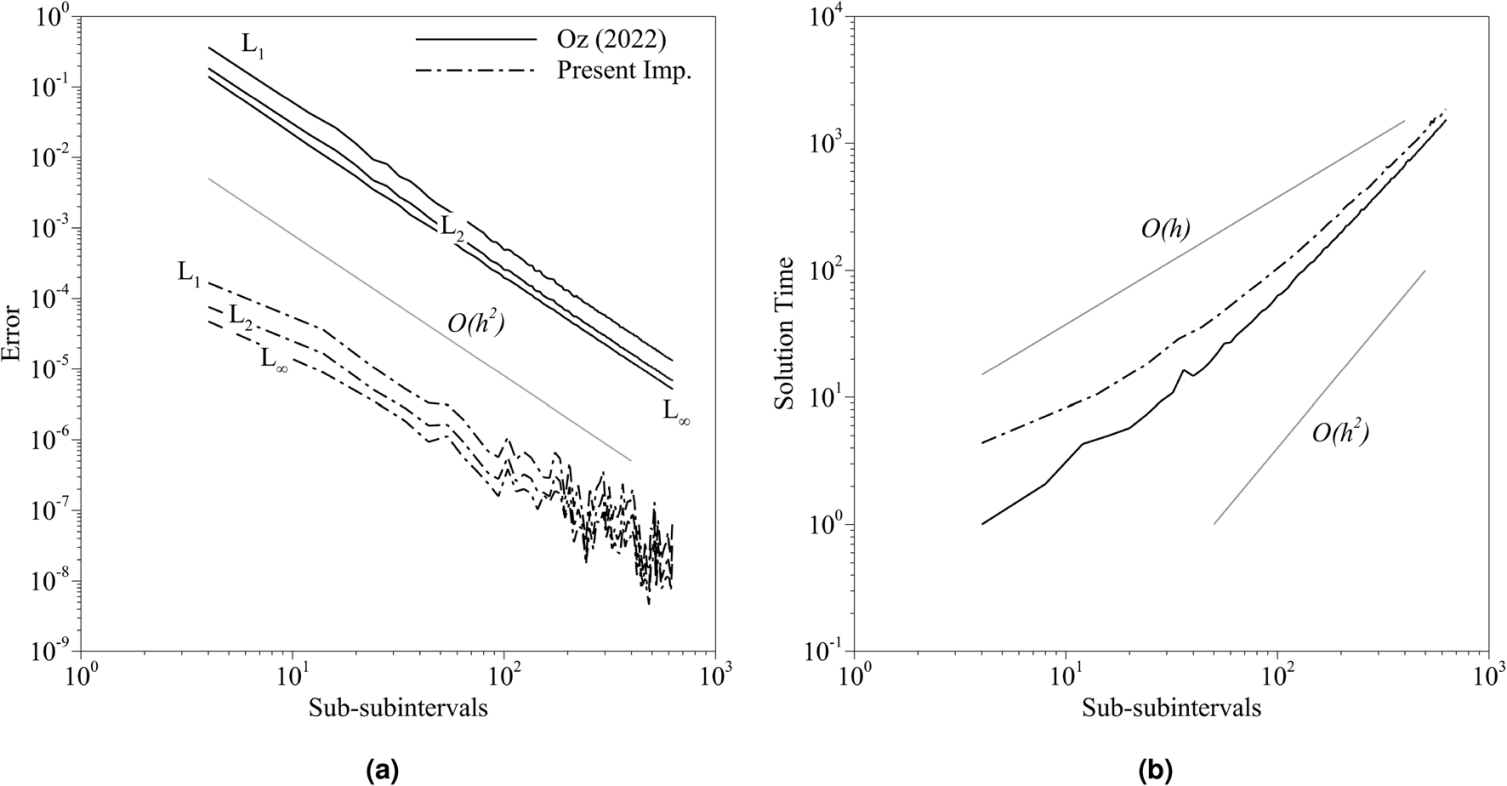
The distribution of the (**a**) errors and (**b**) normalized solution times obtained from the carried out simulations for both implementations with 5 subintervals where the axes are logarithmic.

In the present implementation, $K_{ns}$ will be taken as 50, 000. First, one of the free parameters has to be constant to examine the other parameter. To that end, the number of subintervals *n* is taken as 5, and the number of sub-subintervals is changed in the range of $[6,\;630]$. The range starts from the low number of sub-subintervals and increases up to the value that is calculated in the previous test case. Figure 5 shows the errors and normalized solution times calculated for both implementations. Both of the plot axes are logarithmic, and the normalization of the time is done by the solution time of the implementation used in the quantum BE solver with 6 sub-subintervals. Thus the solution time plot starts from 1 for the old implementation. The errors are decreasing with the increasing sub-subintervals, as expected for all error types. The order of the error scales with the square of the time spacing as the parameter *r* is taken as 2. However, the error amplitude in the present approach significantly differs from the old method. Figure 5a shows that the old implementation reached up to the order of $O(10^{-5})$ with the highest number of sub-subintervals used in this study. The same order of accuracy is reached with approximately 10 sub-subintervals. However, at the maximum number of sub-subintervals used in the study, the accuracy reached $O(10^{-9})$. Moreover, the old implementation reached the order of $O(10^{-5})$ while the present implementation reached up to the order of $O(10^{-9})$ at the approximately same time (Fig. 5b). The old implementation reached the order of $O(10^{-5})$ accuracy approximately 100 times faster. The reason for the gain in the amplitude of the error is because of the increased number of sampling points used in the QAEA, which leads to better accuracy. It is also observed that oscillations appear after a specific limit with the new implementation. Our initial observation shows that the probabilistic behavior of QAEA causes these oscillations. However, it is still not clear, and it requires in-depth investigation. As a result, we leave these oscillations as the subject of future studies.

**Figure 6:**
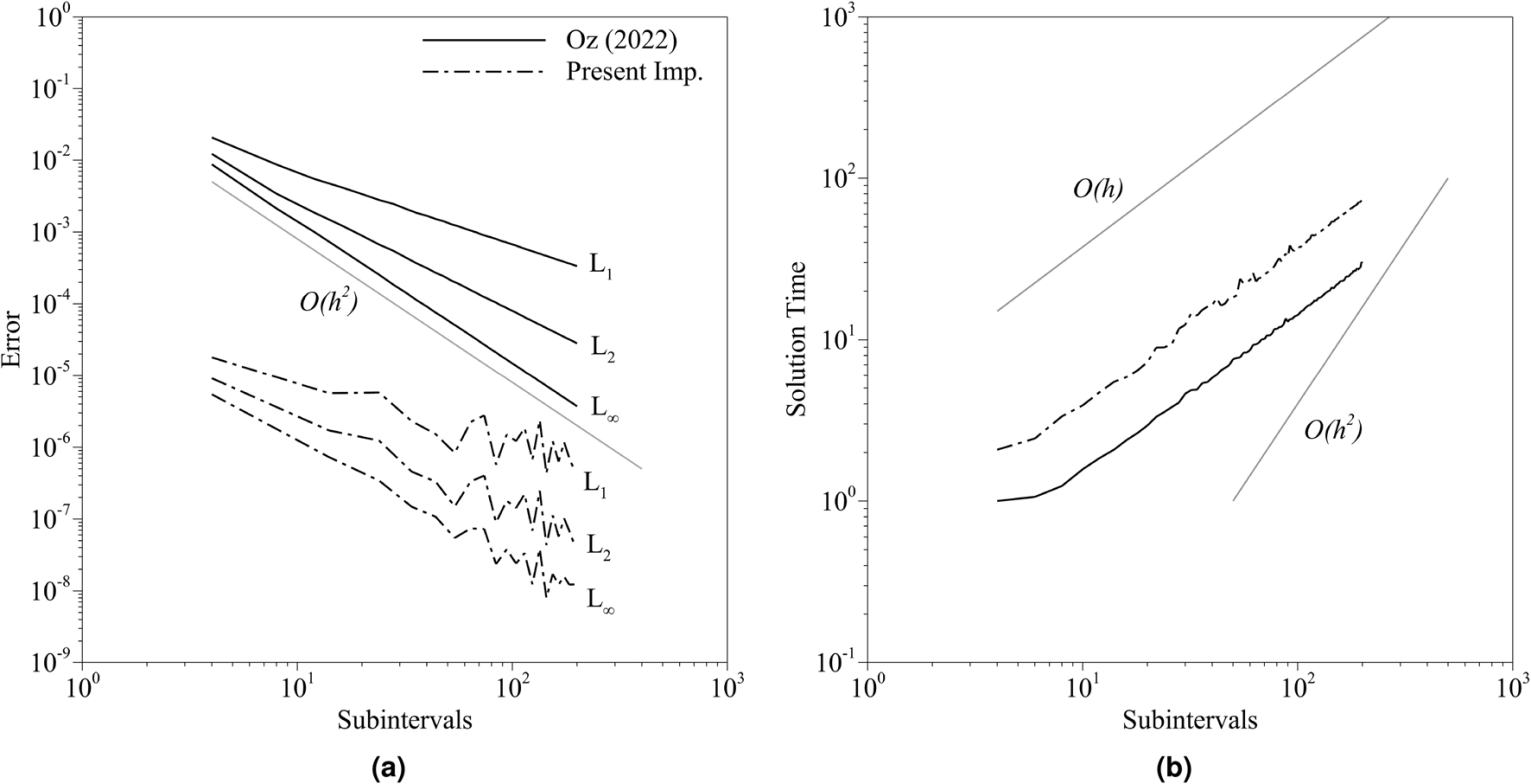
The distribution of the (**a**) errors and (**b**) normalized solution times obtained from the carried out simulations for both implementations with 20 sub-subintervals where the axes are logarithmic.

Lastly, the number of sub-subintervals will be constant, and the number of subintervals *n* will be taken as a free parameter, and it will change in the range of $[6\;200]$. The number of sub-subintervals is specified as 20. Our experience indicates that other selections lead to similar observations. Thus the simulations with $K_{nf}$ 20 will be provided. $K_{ns}$ will be taken as 50, 000. Figure 6 shows the errors and normalized solution times calculated for both implementations. Both of the plot axes are logarithmic, and the normalization of the time is done by the solution time of the implementation used in the quantum BE solver with 6 subintervals. The error and solution time plots yielded similar results with the variable sub-subinterval case. However, the old implementation leads to wider error distribution with increasing subintervals. L$_\infty$ is decreasing more than L$_1$ error, which indicates that the error distribution is getting close to uniform distribution because the total error in the solution vector is decreasing more than the cumulative error in the solution vector. It may be due to the fact that errors introduced by the number of subintervals saturate slowly. Hence, the error introduced by the other sources starts to dominate. As a result, the error distribution is uniform because of the other error sources as the number of subintervals decreases. For the solution time, it is possible to observe a similar trend to the previous case. At the time old implementation achieved $O(10^{-5})$ L$_2$ error accuracy, the new implementation reached $O(10^{-7})$. Additionally, in this test case, the old implementation reached a maximum order of $O(10^{-5})$ L$_2$ error accuracy. However, the lowest L$_2$ error accuracy is in the order of $O(10^{-6})$ with the present implementation, and this accuracy is achieved approximately 0.07 times faster.

### Heat equation

Herein, the ODE problem is extended to the PDE problem. The first PDE problem is defined as in Eq. (24), where the analytical solution is given as Eq. (29). The given equation includes the diffusion (viscous) term in the Navier–Stokes equation, which is critical in the aerospace industry and various other fields, as mentioned by References^19,59,60,88–91^. In PDEs, discretization in space is required. As a result, the PDE can be written as a system of ODEs, as shown in Eq. (1). Inherently, the error calculated in this subsection will include spatial discretization error. However, it will be minimized by using high-order methods.

**Figure 7:**
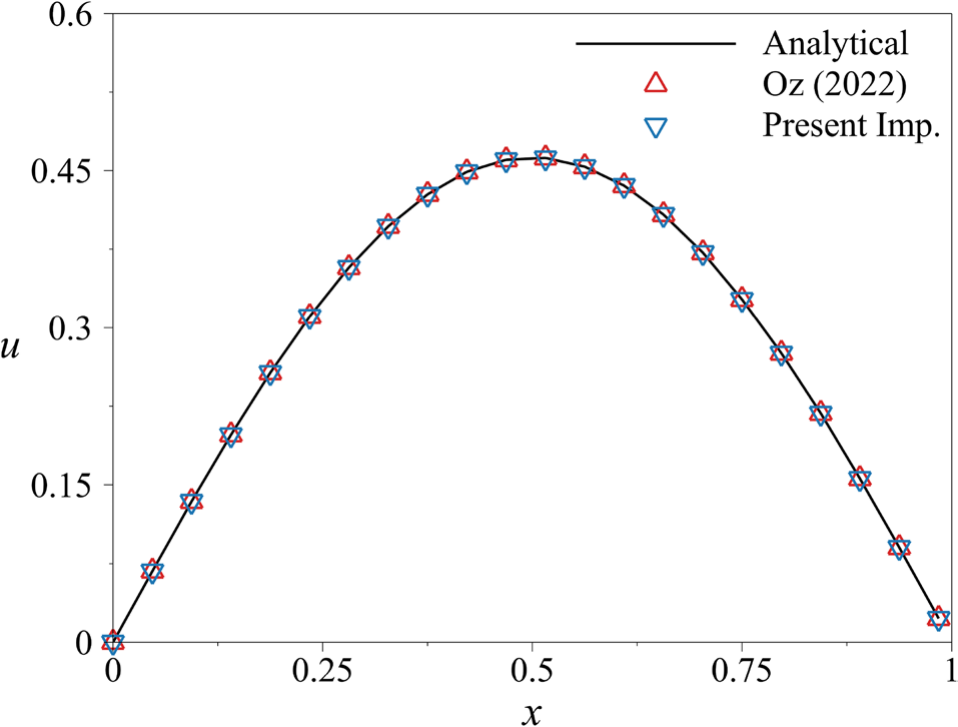
The comparison of results obtained with quantum PDE solver using two implementations and the analytical solution in the interval of $T=[0\;0.078125]$ with $n=300$. Every third point is shown for clarity.

First, the boundary conditions of the system are given as $u(0,t)=0$ and $u(L,t)=0$, where *L* is the length of the computational domain, and it is taken as $L=1$. The initial condition of the system is $u(x,0)=\sin (\pi x)$. The simulations will be carried out with 65 lattice points with the fourth-order finite difference method in the interval of $T=[0\;0.078125]$ and thermal diffusivity $\alpha ^2=1$. Initially, the equation is solved with the CFL condition where the *CFL* number is taken as 0.1. With the given conditions, the parameters are calculated as $n=300$ and $N_k=300$. For a better comparison, the same number of subintervals are used with the present implementation along with $K_{nf}=10$. The free parameter $K_{ns}$ is taken as 920. This value is much lower than the one used in the ODE problem. Our experience shows that in PDE problems, lower $K_{ns}$ than the ODE problem leads to better accuracy. Otherwise, it leads to unnecessary calculations, which help spatial error to cumulate. Figure 7 shows the solution vector obtained at the end of the simulations. The figure shows every third point of the solution vector for clarity. In the results, there is no distinguishable difference. The dissipative behavior from the second-order derivative decreased the maximum amplitude of the initial wave, and the quantum PDE algorithm solved it with great accuracy.

The detailed error analysis is provided in Table 2. Unlike the ODE case, the accuracy of the old implementation is as high as the present implementation. However, the solution time is approximately 40 times higher for the old implementation. L$_1$ error is higher than the L$_2$ error, which is expected to observe because of the definition of the errors. L$_\infty$ error, on the other hand, is so close to the order of $O(10^{-9})$ for both of the implementations.

**Table 2:** The normalized solution times and errors obtained by the quantum PDE algorithm for two different implementations. Although accuracy gain is not observed, the solution time decreases approximately 40 times with the new implementation.

	Solution	L$_1$	L$_2$	L$_\infty$
	Time	Error	Error	Error
Oz^19^	1	$5.6989\times 10^{-7}$	$8.1340\times 10^{-8}$	$1.5865\times 10^{-8}$
Present Imp.	0.024	$6.3932\times 10^{-7}$	$8.2208\times 10^{-8}$	$1.3419\times 10^{-8}$

**Figure 8:**
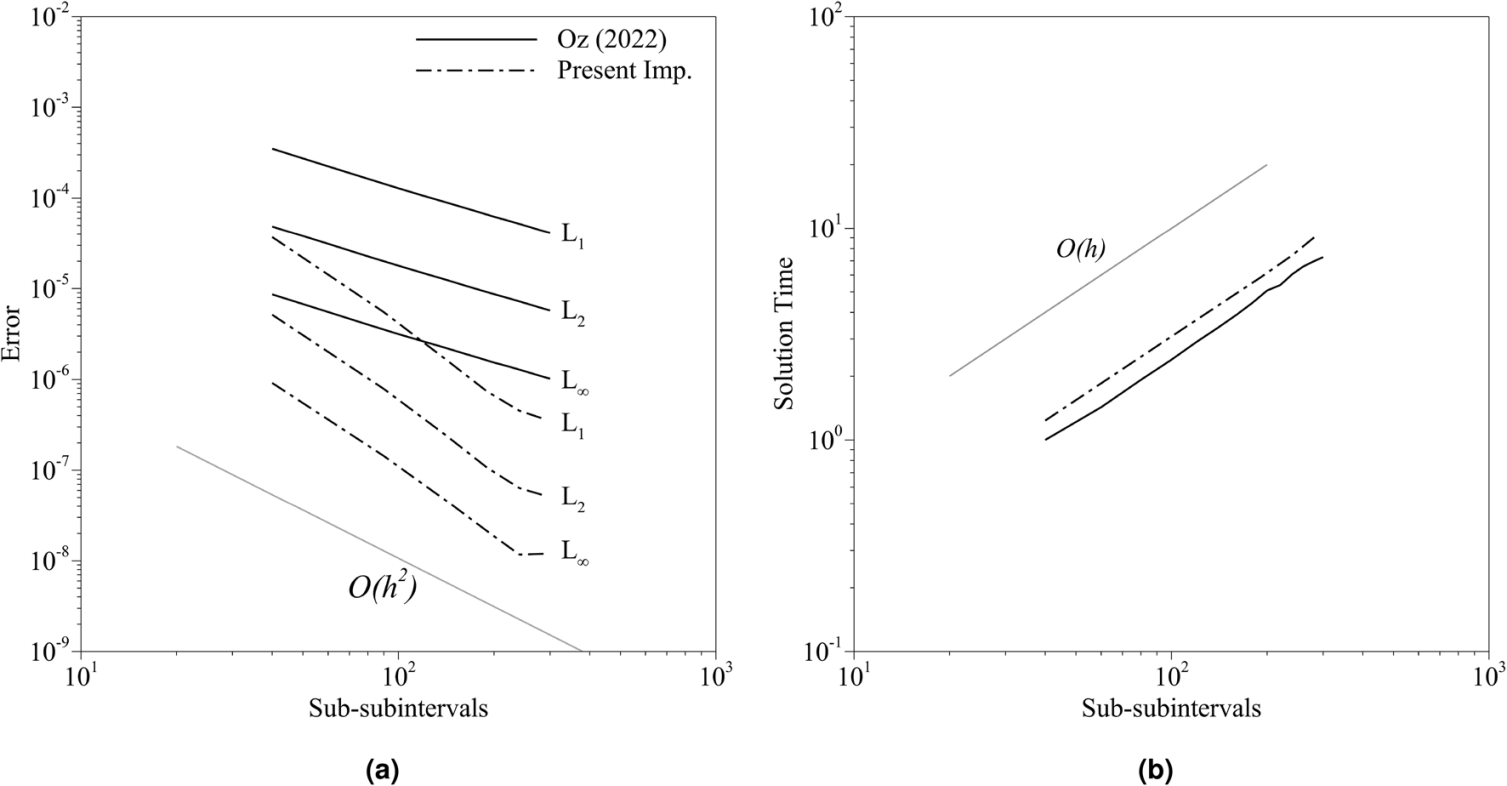
The distribution of the (**a**) errors and (**b**) normalized solution times obtained from the carried out simulations for both implementations with 30 sub-subintervals where the axes are logarithmic.

In the heat equation, the number of subintervals is more important than the ODE problem because of the nature of PDEs. Thus, the performance of both implementations will be investigated with a free parameter for the number of subintervals with a constant number of sub-subintervals. In this study, the number of sub-subintervals is specified as 30. Although it is lower than what is obtained, when the conditions are enforced, it will show the error trend with the changing number of subintervals in the range of $n=[30,\;300]$. When the number of subintervals is lower than 30, the algorithm fails to converge due to the stability condition for both implementations. Figure 8 shows the error and normalized solution time of the carried-out simulations. The normalization is done by the old implementation with $n=30$. Both axes are logarithmic to show the error slope and solution times. Same as the ODE case, the second-order accuracy was expected. However, in the proposed method, the slope of the error is slightly steeper than the second order. The reason might be the cancellation of the higher order error terms with the proposed method. As a result, the error from higher-order terms is smaller than the other test cases used in the study. The reason the same behavior is not observed with the old implementation may be the other factors contributing to the error, such as an error coming from QAEA. The deviation from the second order with increasing sub-subintervals in the proposed method indicates additional error sources affecting the overall error (Fig. 8a). The error is drastically decreasing with the present implementation. The present implementation’s errors start to decrease when the new implementation reaches the lowest error within the test case intervals. L$_\infty$ error of the old implementation starts from in the order of $O(10^{-5})$ and reaches up to order of $O(10^{-6})$ (Fig. 8a). However, the present implementation starts from the order of $O(10^{-6})$ and reaches up to the order of $O(10^{-8})$. The slope of the errors is different in the two implementations. In the ODE case, the slope of the error is approximately constant for both implementations. The reason might be because of the spatial error. In the old implementation, the error coming from the quantum algorithm was higher. In every subinterval, spatial discretization will introduce additional errors. Thus this will accumulate in every subinterval. Although the slope of the errors is different in both implementations, they are the same in the solution time plot (Fig. 8b). The ratio of the fastest solution of the present implementation to the slowest solution of the present implementation is 0.17. The present implementation is approximately 5 times faster than the old implementation within the test case limits.

### Convection–diffusion equation

**Figure 9:**
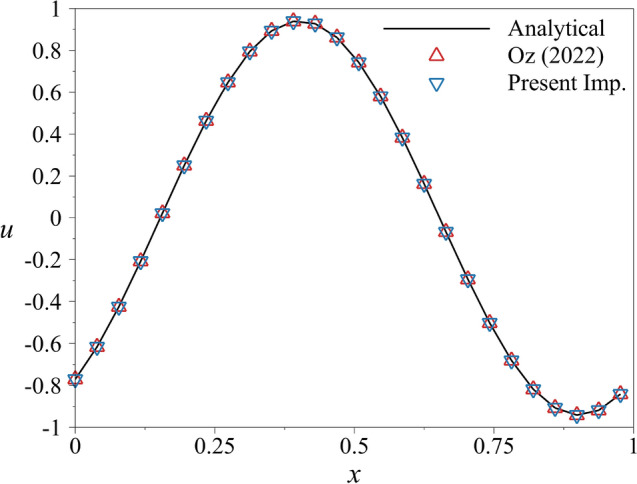
The comparison of results obtained with quantum PDE solver by using two implementations along with the analytical solution in the interval of $T=[0\;0.015259]$ with $n = 70$. Every tenth point is shown for clarity.

Herein, we extend our previous heat equation case into a more complex PDE where both convective and diffusive terms are included. The partial differential equation is defined in Eq. (33), where the analytical solution is defined as shown in Eq. (35). The problem is a simplified version of the Navier-Stokes equations which is crucial in the aerospace industry. The aforementioned spatial error included in the error norms will be observed in this case as well. To minimize it, high-order spatial discretization, namely the fourth-order central scheme, is used in this test case as shown in Eqs. (36–40). The thermal diffusivity $\alpha ^2$ is taken as 0.1 while the velocity of the wave, *c* is taken as 10. This combination ensures that the wave is moving by losing its amplitude slowly. The computational domain is limited to $x=[0\;1]$ where the initial condition is $u(x,0)=\sin (2\pi x)$. It has to be noted that the boundary conditions used in this test case are periodic boundary conditions which make the limits of Eq. (1) in the range of $1\le j\le m$ because of the boundaries included in solution vector. In the simulation, the number of subintervals is specified as 70. Conditions enforced to determine the number of sub-subintervals led to 4900. The same number of subintervals is used with the present implementation. However, $K_{nf}$ is taken as 500 and $K_{ns}$ is taken as 3000. A new case is also simulated with the present implementation where the number of subintervals is 700, the $K_{nf}$ is 30, and $K_{ns}$ is 3000. The present implementation requires a low number of sub-subintervals for better performance. The additional computational cost introduced by the new implementation can be neglected by the low number of sub-subintervals. However, 500 sub-subintervals lead to longer solution times with less accurate results. It has to be noted that 700 subintervals lead to 490, 000 sub-subintervals. Thus, the simulation is not run for $n=700$ with the old implementation. In this problem, the number of elements used in spatial discretization is 264. Lower values led to spatial error-dominated results, which prevented observing an advantage with the current implementation. The simulations are carried out by both of the implementations, and the results are shown in Fig. 9. The solution vectors of both implementations coincide with the analytical solutions as the error of the solution vectors are close to machine zero. The figure shows every tenth point to avoid a complex-looking figure.

The details of the error norms, along with the normalized solution time, are given in Table 3. The time normalization is done with the old implementation. The table shows that the accuracy of the previous implementation is close to machine zero. However, the proposed implementation with $n=70$ leads to one order higher L$_2$ and L$_\infty$ errors along with two orders higher L$_1$ error. Although the solution time is 8 time faster, the accuracy is lower than the old implementation. The advantage of the old implementation can be observed with the higher number of subintervals, where the number of sub-subintervals can be lower. In regular computational simulations, the number of subintervals increment should lead to, generally speaking, higher solution times. However, in the present implementation, increasing the number of subintervals, *n*, does not increase the solution time because of the decreasing number of sub-subintervals. When $n=700$, the present implementation provided the same accuracy in a 20 times faster solution time. Additionally, if we consider the solution time gain as the complexity of the problem increases (ODE, heat equation, convection–diffusion equation), the solution time gain decreases with the complexity. The decrease in solution time gain is because of the increase in lattice points. Increasing the lattice point leads to an increase in the number of ODEs in the system. Thus the function given in Eq. 9 has to be written for every ODE in the system. Although it is not an expensive process, it contributes to the total time with the high number of spatial discretization points. Additionally, an increased number of sub-subintervals required to obtain specific order of accuracy contributes to total time, yet the solution time gain is still high. Quantitatively, an algorithm providing the solution 20 times faster will provide the results of a day-long simulation in 1.20 hours. It is important to note that the simulation times may change when the quantum algorithm is run on a real quantum computer. The calculation of the average value of the driver function appearing in Eq. (4) by the QAEA requires some time. Although it is shown that QAEA may lead to a quadratic speedup^19,59,60^, it requires quantum computers to observe the speedup. The numerical simulation of the quantum algorithm on a classical computer is not expected to show a quantum speedup as classical computers do not generate the quantum entanglement or state superposition that underlies the speedup. Nonetheless, in this paper, QAEA is simulated under the same conditions for both implementations. Thus, even if QAEA shows a quantum speedup on a quantum computer, the solution time gain obtained by the present implementation should be observable.

**Table 3:** The normalized solution times and errors obtained by the quantum PDE algorithm for two different implementations with $n=70$ and $n=700$.

	Num. of	Solution	L$_1$	L$_2$	L$_\infty$
	Subint.	Time	Error	Error	Error
Oz^19^	70	1	$4.4797\times 10^{-7}$	$3.1025\times 10^{-8}$	$2.7335\times 10^{-9}$
Present Imp.	70	0.123	$1.2151\times 10^{-5}$	$8.4202\times 10^{-7}$	$7.4375\times 10^{-8}$
Present Imp.	700	0.047	$6.0950\times 10^{-7}$	$4.2291\times 10^{-8}$	$3.7378\times 10^{-9}$

**Figure 10:**
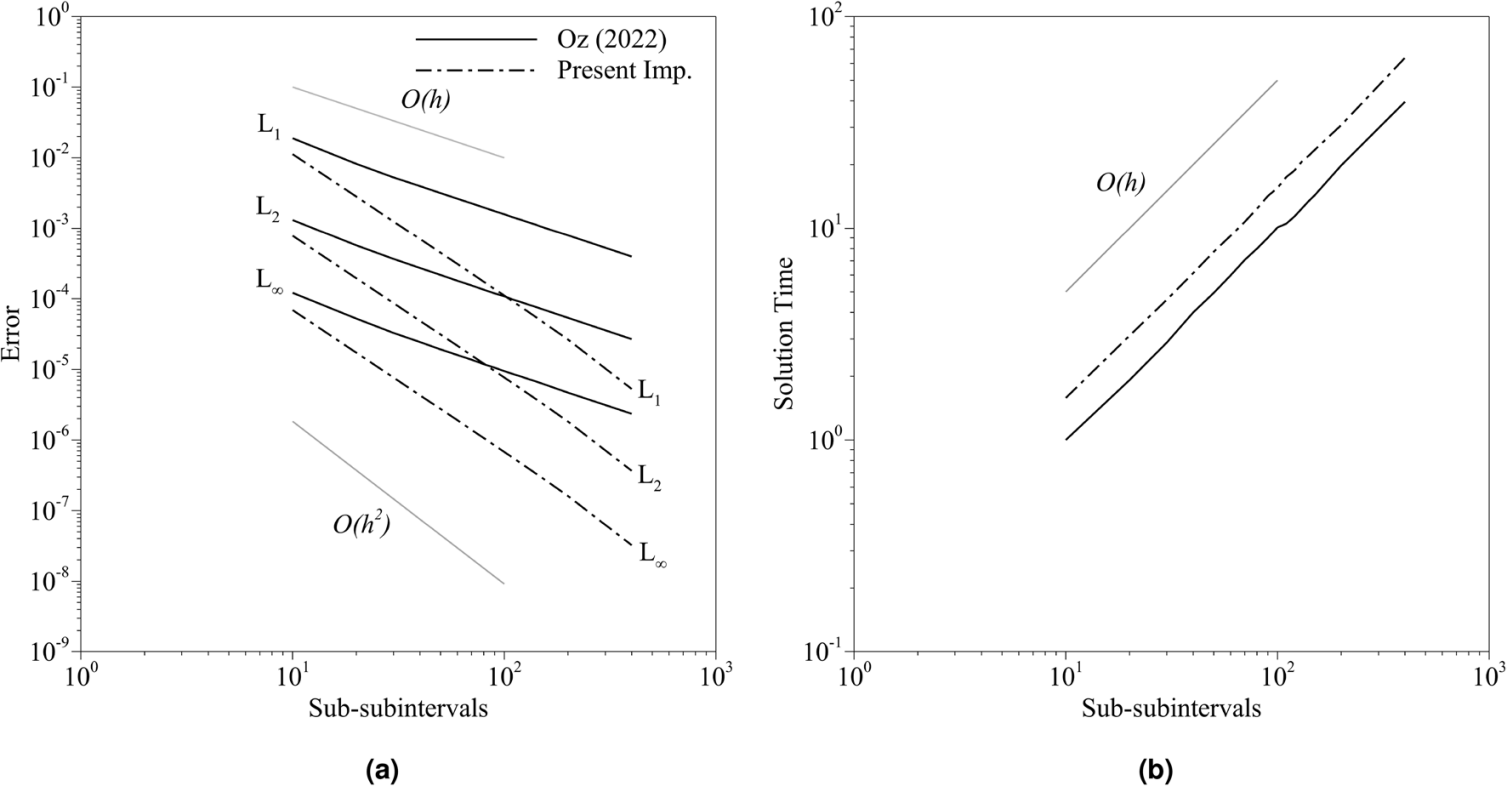
The distribution of the (**a**) errors and (**b**) normalized solution times obtained from the simulations for both implementations with 30 sub-subintervals where the axes are logarithmic.

Table 3 shows the error norms and solution times with a certain number of subintervals, but the development of the error norms with the number of subintervals is not investigated. In the following simulations, the number of sub-subintervals is initially specified as 30 for both implementations. The number of subintervals, *n*, is specified as a free parameter that will be changed. The parameter $K_{ns}$ is chosen as 3000. The number of subintervals is changed in the range of $n=[10,\;400]$. The norms and normalized solution times are given in Fig. 10. The normalization is done with the solution time obtained by the old implementation with $n=10$. The error slope for the present implementation is higher than the old implementation (Fig. 10a). It must be noted that the number of sub-subintervals in the old implementation must be calculated with the aforementioned conditions. However, it is chosen as a certain number to compare under the same conditions. Yet, Table 3 shows the results with the conditions enforced. Although the expected order of accuracy is two, the old implementation led to lower orders because of the contribution coming from the quantum algorithm. The proposed algorithm, however, is closer to the second order. At the same time, the error amplitude is also lower. The present implementation’s simulation time is approximately 1.5 times higher with the same number of subintervals, yet it provides two-order more accurate results. The old implementation leads to $O(10^{-5})$ accuracy (considering L$_2$ norm) with $n=400$. The same order of accuracy is obtained with $n=60$ with the current implementation. Moreover, the solution vectors are 4.3 times faster with the proposed implementation. In the present implementation, specifying optimum $K_{nf}$ and $K_{ns}$ parameters is important. $K_{ns}$ may change drastically depending on the function. Although using high values for $K_{ns}$ will not affect the results, it may lead to increased solution times. The optimum selection of $K_{ns}$ requires experience. For an optimum starting point, we suggest to define $K_{ns}$ as a square of $K_{nf}$ where $K_{nf}$ is mostly limited by the physical quantities. Based on the desired order of accuracy, $K_{ns}$ can be gradually increased until the targeted order of accuracy is achieved.

## Conclusion

As quantum computer and quantum algorithm developments are accelerating, the pursuit of a way to implement efficient quantum algorithms for quantum computing is rising. Researchers from different fields are looking for alternative methods to solve their physical problems that are not feasible to solve in classical computers. This paper proposes an efficient implementation with Chebyshev points for the quantum PDE algorithm developed by Gaitan^59,60^. Simulations are carried out for three different equations: a generic ODE, heat equation, and convection–diffusion equation. The analytical solutions of the equations are used to calculate the error of the solution. The L$_1$, L$_2$, and L$_\infty$ norms of the error vectors are obtained for the proposed implementation and compared with the literature. The results showed that the proposed implementation might lead to a two-order accuracy gain and up to 100 times solution time decrease. Although the results of the test cases in this study indicated a significant speed-up with the proposed algorithm, the value of the approach depends on the complexity of the problem, such as PDEs with both convection and diffusion terms. With the additional physical complexity, the speed-up advantage of the algorithm is reduced. Thus, we will explore the quantum advantage of our approach in high-dimensional settings and more complex problems. Moreover, the present implementation of our approach requires the definition of several user-defined parameters, such as $K_{n,s}$ and $K_{n,f}$. These hyperparameters can be automated from the temporal dynamics of the underlying problem, a topic we would like to explore in the future. We also plan to use the proposed algorithm in real quantum machines. Finally, we would like to conclude that quantum computing is still in a very early stage, and researchers working at the interface of quantum computing and numerical methods will significantly contribute to this emerging field.

## Data Availability

The datasets of the current study are available from the corresponding author upon request.
